# A systematic review of ethical considerations of large language models in healthcare and medicine

**DOI:** 10.3389/fdgth.2025.1653631

**Published:** 2025-09-11

**Authors:** Muhammad Fareed, Madeeha Fatima, Jamal Uddin, Adeel Ahmed, Muhammad Awais Sattar

**Affiliations:** ^1^ Riphah School of Computing & Innovation, Riphah International University, Islamabad, Pakistan; ^2^ Department of Computer Science, Electrical and Space Engineering, Luleå University of Technology, Luleå, Sweden

**Keywords:** artificial intelligence (AI), deep learning, large language models (LLMs), ChatGPT, bioethical issues, bias, fairness, privacy

## Abstract

The rapid integration of large language models (LLMs) into healthcare offers significant potential for improving diagnosis, treatment planning, and patient engagement. However, it also presents serious ethical challenges that remain incompletely addressed. In this review, we analyzed 27 peer-reviewed studies published between 2017 and 2025 across four major open-access databases using strict eligibility criteria, robust synthesis methods, and established guidelines to explicitly examine the ethical aspects of deploying LLMs in clinical settings. We explore four key aspects, including the main ethical issues arising from the use of LLMs in healthcare, the prevalent model architectures employed in ethical analyses, the healthcare application domains that are most frequently scrutinized, and the publication and bibliographic patterns characterizing this literature. Our synthesis reveals that bias and fairness (n=7, 25.9%) are the most frequently discussed concerns, followed by safety, reliability, transparency, accountability, and privacy, and that the GPT family predominates (n=14, 51.8%) among examined models. While privacy protection and bias mitigation received notable attention in the literature, no existing review has systematically addressed the comprehensive ethical issues surrounding LLMs. Most previous studies focus narrowly on specific clinical subdomains and lack a comprehensive methodology. As a systematic mapping of open-access literature, this synthesis identifies dominant ethical patterns, but it is not exhaustive of all ethical work on LLMs in healthcare. We also synthesize identified challenges, outline future research directions and include a provisional ethical integration framework to guide clinicians, developers, and policymakers in the responsible integration of LLMs into clinical workflows.

## 1 Introduction

Artificial intelligence (AI) aims to give computer systems cognitive abilities akin to those of humans through tasks such as perception, reasoning, and decision-making (1, 2). A powerful subset of AI, deep learning employs multi-layer neural networks to extract hierarchical features from large datasets automatically (3). One significant advancement in deep learning is the transformer architecture, which relies on self-attention mechanisms (4). This architecture enables efficient parallel processing and captures long-range dependencies, revolutionizing sequence modeling (5). The recent rise of large language models (LLMs) depends on transformers and involves pre-training on extensive text corpora, followed by optimization for specific tasks (6). Notable examples of these models include Claude from Anthropic (7–10), Google’s Bard/Gemini (8–10), Meta’s LLaMA family (11–13), Google’s BERT and its derivatives (14–17), and OpenAI’s GPT series (such as GPT-3.5 and GPT-4) (8, 10, 12, 18–27). These LLMs excel at producing coherent text, summarizing complex documents (28), and engaging in multilingual conversations. Their application in healthcare, including clinical decision support (29) and chatbots that interact with patients (30), indicates a significant shift in how medical knowledge is accessed and utilized. However, using LLMs in clinical settings raises important ethical concerns (31). Bias in training data can lead to unfair outcomes (27), and the “black-box” nature of these models makes decision-making processes opaque (14, 21). Concerns about patient data privacy (12, 14, 23) also arise, along with risks of misuse, such as generating harmful or misleading medical advice (11, 18). It is crucial to address these ethical issues to ensure that innovations driven by LLMs enhance patient safety (25), promote equity (18), and build trust (11, 19–22, 25, 32) within healthcare systems.

The motivation for this survey arises from the rapid proliferation of large language models (LLMs) in healthcare and the recognition that existing reviews have significant shortcomings. Prior studies often reveal inconsistent evaluation methods, biases, and underrepresentation of medical-domain LLMs, along with limited or heterogeneous ethical analysis and a lack of standardization in literature selection (33). Many reviews inadequately address non-binary identities, potential publication bias, or regulatory frameworks. They frequently omit explicit measurement guidelines or in-depth policy discussions and treat LLMs only briefly, with cursory mentions of tools like ChatGPT (34–36). Some reviews focus narrowly on technical aspects or single specialties without systematic methodology, empirical depth, or broader healthcare ethics. Others rely on short timeframes or preprint sources, which reduces generalizability (37–41). Consequently, there is a clear need for a rigorously conducted, up-to-date synthesis that systematically evaluates the ethical contributions, challenges, and governance considerations of LLM deployment across diverse healthcare contexts.

A systematic literature review was conducted following the guidelines of the Preferred Reporting Items for Systematic Reviews and Meta-Analyses (PRISMA) 2020 (42) and the recommendations from Kitchenham & Charters (2007) (43), ensuring methodological rigor and transparency. This review proceeded through four phases: (1) a preliminary study to define research questions and identify relevant search terms; (2) a screening process in which 316 records retrieved from the ACM Digital Library, SpringerLink, Wiley Online Library, and PubMed (spanning 2017 to 2025) were screened based on their titles and abstracts, followed by deduplication; (3) an eligibility and quality assessment that applied predefined criteria to the full texts, resulting in 27 primary studies specifically addressing the ethical considerations of large language models in healthcare; and (4) data extraction and compilation, which involved capturing bibliographic details and ethical-specific variables.

This review aims to provide practical guidance for clinicians, developers, and policymakers and to chart clear directions for the responsible integration of LLMs in healthcare. The primary objectives of this study are to:

• Identify the main ethical issues arising from LLM deployment in healthcare.
• Survey on which model architectures are most frequently employed in ethical analyses.
• Map the healthcare application domains that receive the most significant ethical scrutiny.
• Examine publication and bibliographic patterns in this literature.
• Critically assess how areas such as privacy, bias and fairness, transparency and explainability, accountability and legal considerations, and safety are treated in existing work and pinpoint methodological and conceptual gaps.
• Propose a provisional ethical integration framework for LLMs in healthcare, organizing regulatory, technical, human oversight, and accountability dimensions.
• Synthesize the ethical contributions and recommendations of prior studies, systematically mapping these dimensions to reveal shortcomings and guide future inquiry.

The structure of this review is as follows: Section 1 presents the rise of large language models in healthcare, defines the scope and objectives, and formulates research questions targeting ethical contributions, policies, challenges, and future directions. Section 2 provides foundational concepts in AI ethics and LLMs, including key dimensions such as privacy, bias/fairness, explainability, accountability, and governance, and outlines the relevant regulatory and framework landscape. Section 3 summarizes existing surveys on AI ethics in healthcare and highlights gaps in systematic, LLM-focused ethical reviews. Section 4 describes the four-phase PRISMA- and Kitchenham-guided approach: initial keyword identification and research question formulation, screening, eligibility and quality assessment, and data extraction. Section 5 synthesizes key ethical findings for each research question with tables and figures. Section 6 examines challenges, proposes an ethical framework, and suggests future research and policy directions. Finally, Section 7 distills main takeaways, research gaps, and practical guidance for responsible LLM deployment in healthcare.

## 2 Preliminaries

### 2.1 Overview of large language models

**Figure 1:**
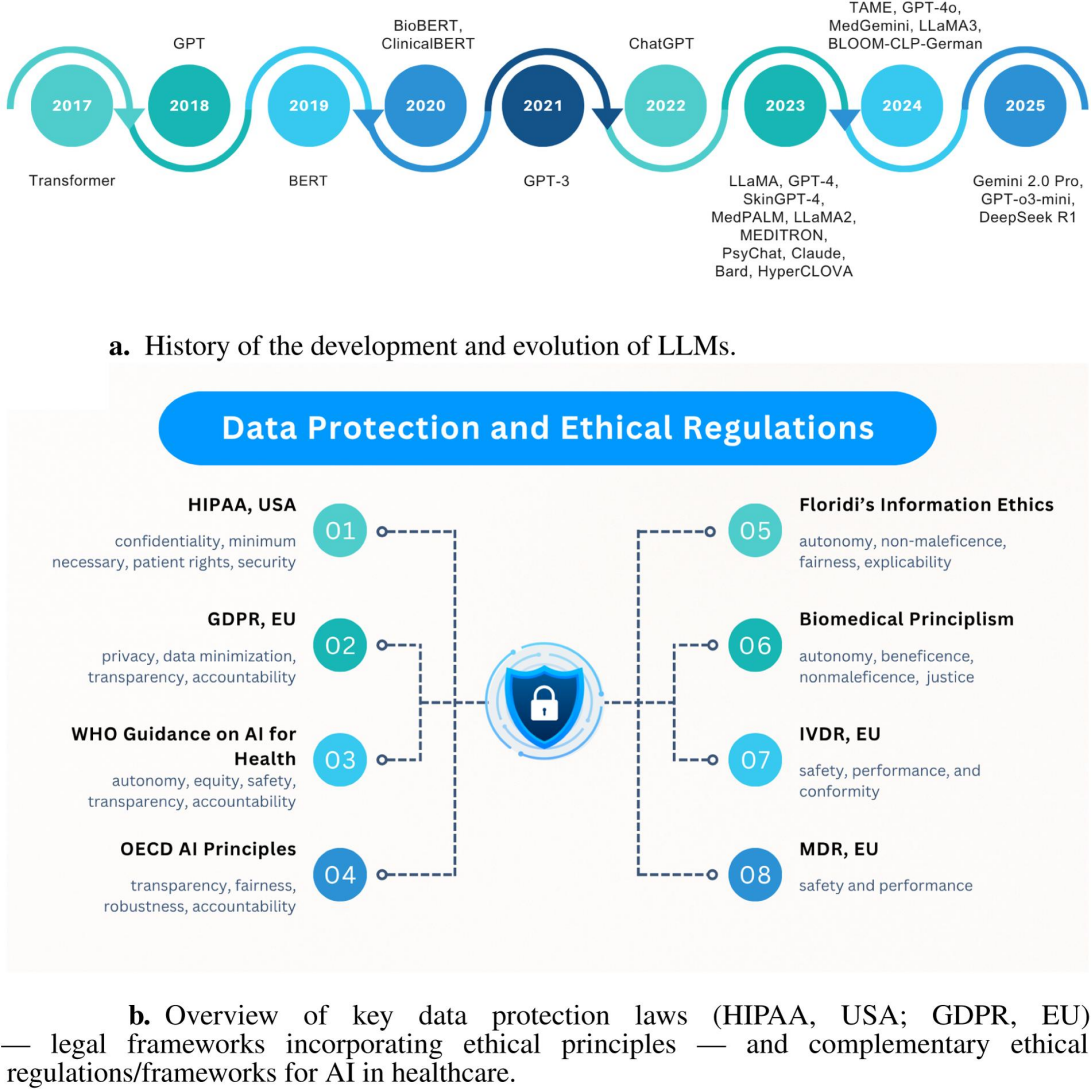
Illustrations of key background concepts. **(a)** History of the development and evolution of LLMs. **(b)** Overview of legal frameworks and ethical regulations of AI in healthcare.

Large Language Models (LLMs) are advanced neural networks trained on vast textual data to understand and generate human-like language, forming the basis for healthcare applications such as summarization, question answering, and decision support (44). From 2017 to 2025, LLM development has followed a clear trajectory: starting with the Transformer architecture in 2017 (45), followed by GPT in 2018 (20) and BERT in 2019 (46). Specialized variants like BioBERT (47) and ClinicalBERT (48) emerged in 2020. GPT-3 (49) appeared in 2021, and the release of ChatGPT in late 2022 sparked broader medical experimentation. In 2023, models such as LLaMA (50), GPT-4 (51), SkinGPT-4 (52), MedPALM (53), LLaMA2 (50), MEDITRON (54), PsyChat (55), Claude (56), Bard (57), and HyperCLOVA (58) tailored to medical data emerged. In 2024, further domain-specific LLMs like TAME (59), GPT-4o (60), MedGemini (61), LLaMA3 (62), and BLOOM-CLP-German (63) continued this trend. Anticipated releases in 2025, such as Gemini 2.0 Pro (64), GPT-o3-mini (65), and DeepSeek R1 (66), promise deeper clinical integration. This evolution, illustrated in Figure 1a, shows the history and evaluation of LLMs.

#### 2.1.1 Generative pretrained transformer (GPT) models

GPT models, such as GPT-3.5 and GPT-4 (8, 10, 12, 18–27), are decoder-only transformer-based generative systems trained on extensive text corpora. They are commonly used in healthcare for drafting clinical notes, summarizing records, and creating decision-support tools. Although these models generate coherent text, they risk producing “hallucinations”—plausible yet incorrect medical statements—requiring human oversight (11, 22). Biases in training data can disadvantage underrepresented patient groups (25). Their proprietary nature complicates Openness and trust (9, 21, 32). Fine-tuning with sensitive data demands strict de-identification and access controls to prevent privacy leaks (12, 20, 23). Integrating GPT models into clinical workflows may slow efficiency due to necessary verification steps, while regulatory uncertainties pose legal and liability challenges. Additionally, their computational demands may restrict real-time deployment without effective tuning strategies.

#### 2.1.2 Bidirectional encoder representations from transformers (BERT)

Encoder-only transformers like BERT, BioBERT, and ClinicalBERT (14–17) excel at entity extraction, coding, and classification but do not generate text. However, they carry ethical risks, as latent biases in embeddings can lead to unfair classification outcomes across demographic groups (15, 17). Their opaque decision logic complicates justifying automated recommendations to clinicians (14, 16). Fine-tuning electronic health records requires careful data handling to prevent misinterpretation of sensitive details. Deploying BERT variants in healthcare often needs domain-specific adaptations, like medical tokenization, along with transparent audit processes to meet regulatory standards. Although smaller than large generative models, they benefit from efficient fine-tuning methods suited for limited hardware in many clinical settings (16).

#### 2.1.3 Large language model meta AI (LLaMA) models

LLaMA models (11–13) provide moderate-size transformer architectures suitable for community fine-tuning under various licensing terms. Their open-source nature promotes clarity but raises misuse concerns (13). Pre-training on general corpora may misalign with medical contexts unless finely tuned, risking biased outputs (13). The absence of a central provider leads to a lack of accountability, requiring institutions to establish their own validation processes. Fine-tuning is feasible on smaller hardware but needs annotated medical data. Integrating these models into healthcare systems requires tailored pipelines and rigorous testing for safety and reliability (11).

#### 2.1.4 Claude (Anthropic) models

Claude (7–10), designed for safety-oriented training, aims to minimize harmful outputs and has undergone pilot healthcare examinations. However, the opacity of its training data and safety mechanisms complicates auditing and trust calibration. Despite safety fine-tuning, Claude may still produce subtle errors in medical contexts, necessitating human-in-the-loop checks (10). Access restrictions and costs limit scalability in smaller clinical settings, and using a third-party service in patient care raises data governance and liability concerns that require contractual resolution (8). Thus, while its focus on safety is promising, careful oversight and clear regulatory pathways are needed before clinical deployment.

#### 2.1.5 Bard and Gemini (Google)

These proprietary models (8–10) in Google’s ecosystem assist with tasks like literature summarization and patient FAQs but pose ethical risks. Integrating them into healthcare portals must prevent exposure of sensitive queries and ensure data privacy, while their tendency to “hallucinate” facts introduces misinformation risks. Their internal workings and moderation processes lack documentation, limiting clarity and trust (9). Deployment requires compliance with data protection regulations (e.g., HIPAA, GDPR), sandboxing patient inputs, and fallback strategies for unreliable outputs. Additionally, reliance on external APIs raises concerns about cost, availability, and reliability.

#### 2.1.6 HyperCLOVA

HyperCLOVA, a region-specific LLM trained on local-language corpora (e.g., Korean), enhances linguistic nuance and cultural relevance in healthcare applications (67). However, its focus on a specific population may improve local performance while risking exclusion or misrepresentation of others, raising equity concerns. Regional training data can embed local biases, necessitating evaluation to prevent skewed clinical outputs. The deployment must comply with local data protection and medical regulations, such as Korea’s Personal Information Protection Act (68) and Medical Devices Act (69), requiring tailored compliance workflows. Limited annotated local medical datasets and resource constraints may hinder fine-tuning and validation, highlighting the need for collaborative infrastructure and expertise sharing in adopting HyperCLOVA in healthcare settings.

### 2.2 Overview of key ethical prospects

There are several key ethical concerns regarding the use of large language models in healthcare. These include the importance of providing reliable and safe outputs, safeguarding patient privacy, preventing bias and unfair treatment, and ensuring Explainability and accountability. These foundational definitions pave the way for more in-depth analysis (see Table 1).

**Table 1:** Brief overview of key ethical considerations faced by LLMs in healthcare.

Consideration	Description	Studies
Safety and Reliability	Ensure LLM outputs do not cause harm, behave reliably in clinical contexts, and avoid over-reliance or failures such as hallucinations and patient safety risks.	(20, 22, 24, 70)
Privacy and Security	Protect patient data during model training, inference, and storage. Ensure data sovereignty, access control, de-identification, and confidentiality without sacrificing utility.	(12, 23, 67)
Bias and Fairness	Prevent disadvantages or misrepresentations of demographic and clinical groups in LLM outputs, including biases related to gender, race, neurodiversity, and culture, ensuring equitable performance.	(7, 8, 10, 13, 15, 17, 27)
Transparency and Explainability	Clarify how LLMs generate outputs for clinicians and patients, reducing “black-box” opacity and enabling the detection of evaluation bias.	(9, 14, 21, 32)
Accountability and Legal	Establish mechanisms for responsibility and liability when LLM-driven decisions lead to adverse outcomes, ensuring system integrity and governance.	(25, 26, 31, 71)
Trust and Misinformation/Integrity	Build appropriate trust without overreliance and detect misinformation while maintaining model integrity and user empathy in healthcare.	(11, 16, 72)
Equity and Inclusion	Address power imbalances and health disparities to ensure that LLMs serve diverse populations fairly and inclusively.	(18)
Autonomy and Personalization	Respect patient autonomy by allowing informed use of LLM tools and tailoring outputs to individual needs.	(19)
Legal Liability and Oversight	Clarify legal responsibilities for LLM deployment in clinical workflows, including concerns about care disruption and validation requirements.	(25)

### 2.3 Ethical policies and research ethics protocols

Studies involving LLM-based tools in healthcare adhere to institutional research ethics processes to protect participant welfare and data integrity (7–9, 11, 19, 67). Many investigations secured Institutional Review Board (IRB) approval or exemption before data collection, citing local guidelines [e.g., Ministry of Health and Welfare standards (67), Harvard Medical School IRB exemptions (9), Virginia Tech IRB oversight (7), and other university review boards (8, 19)]. Ethical clearance typically involved de-identification of sensitive data per the Helsinki Declaration, removal of personally identifiable information (11), and informed consent procedures when human subjects were involved (7, 67). In one case, collaboration with a self-advocate advisory committee ensured an inclusive and neuro-affirming study design (7). These protocols show a commitment to research ethics, though many researchers prioritize approval processes over specific AI-related safeguards beyond standard human subjects protection.

### 2.4 Data protection laws and ethical regulations

Positioning HIPAA (US) and GDPR (EU) as illustrative compliance frameworks, explicitly as legal/regulatory instruments that embed ethical principles (e.g., autonomy, transparency), due to their jurisdictional prominence and distinct regulatory philosophies (institutional control vs. individual consent), this analysis views these legal frameworks through an ethical lens rather than providing a legal assessment. Our examination focuses on operational implications for LLM governance, not exhaustive legal interpretation. Beyond these legal instruments, complementary ethical instruments establish baseline deployment principles, with Figure 1b demonstrating how these layered protections collectively enforce patient confidentiality, algorithmic accountability, and clinical safety.

#### 2.4.1 Health insurance portability and accountability act (HIPAA, USA)

HIPAA (73) exemplifies institutional-control approaches through its Privacy Rule limiting protected health information (PHI) use/disclosure, Security Rule mandating technical safeguards, Breach Notification requiring disclosure reporting, and “Minimum Necessary” principle restricting data access. Enforced by the U.S. Department of Health and Human Services Office for Civil Rights with penalty authority, HIPAA imposes specific LLM requirements, including de-identification of training data, encrypted inference pipelines, access controls, and breach detection. Contrasting with GDPR, HIPAA prioritizes institutional stewardship over individual consent.

#### 2.4.2 The general data protection regulation (GDPR, EU)

GDPR (74) represents consent-based governance by mandating lawful processing requirements, such as consent or legitimate interest, and enforcing principles of data minimization, purpose limitation, and storage limitation. It grants data subjects rights to access, rectification, erasure, portability, restriction, and objection, and requires interpretability in automated decision-making by providing clear information about processing logic. EU supervisory authorities may impose significant fines for violations. For LLM applications in healthcare, GDPR requires explicit consent (or another lawful basis) before using patient data, encourages minimal data retention, and obligates data controllers to explain model outputs affecting individuals. Cross-border data transfers must comply with adequacy decisions or contractual safeguards. Diverging from HIPAA’s institutional focus, GDPR centers on individual autonomy.

#### 2.4.3 Other laws, regulations, and ethical frameworks in AI and healthcare

Beyond legal frameworks such as HIPAA and GDPR, numerous international and national instruments guide ethical AI and medical device deployment in healthcare. Notable examples include the World Health Organization's Ethics and Governance of Artificial Intelligence for Health (2021) (76), the U.S. FDA’s AI/ML Software as a Medical Device action plan, and the European Commission’s Ethics Guidelines for Trustworthy AI (2019). Complementing these, recent frameworks like the tripartite responsibility model (75) operationalize bioethical principles by assigning distinct obligations to patients, clinicians, and systems to balance innovation with equitable risk mitigation. These instruments collectively converge on principles of patient safety, fairness, transparency, and accountability. Table 2 maps these laws and guidelines, providing a comprehensive regulatory landscape for LLM integration in clinical contexts.

**Table 2:** Other laws, regulations, and ethical frameworks for AI in healthcare.

Law/regulation	Purpose	Relevance to LLMs in healthcare	Reference
WHO Guidance on AI for Health	Provides principles for trustworthy AI in health and shared decision-making equity	Ensures patient-centered, equitable use of LLMs	(76)
OECD AI Principles	Establishes best practices for AI and human oversight requirements	Informs governance for LLM development and deployment	(77)
Floridi Information Ethics	Outlines ethical principles for information use and epistemic responsibility	Guides ethical evaluation of LLMs in patient applications	(78)
Biomedical Principlism	Frames core biomedical ethics and autonomy preservation	Evaluates LLM impacts on patient autonomy and equity	(79)
In Vitro Diagnostic Medical Devices Regulation (IVDR)	Governs the safety and performance of diagnostic devices	Relevant for LLM-based diagnostic decision-support systems	(80)
Medical Devices Regulation (MDR)	Ensures the safety and performance of medical devices	Applies to LLM-driven software classified as medical devices	(81)
Medical Devices Act No. 15945, 2018	Regulates medical devices in South Korea	Pertinent for LLM-based clinical tools requiring approval	(69)

## 3 Related work

This section examines prior surveys and reviews that address the ethical considerations of large language models in healthcare. It summarizes their scope in clinical use cases and highlights limitations in areas such as bias mitigation, privacy protection, clarity, accountability, and governance. Additionally, it identifies recurring gaps, including inconsistent methodologies, the lack of standardized ethical frameworks, and challenges in scaling oversight mechanisms (see Table 3 for detailed comparisons).

**Table 3:** Summary of prior surveys on ethical considerations of LLMs in healthcare.

Study	Focus Area	Y	D	NPR	P/B	SLR	RSA	SO	ER&F	EPRP	SR	FD
Shool et al. (33)	Clinical Medicine	2019–2025	5	761	–	**	–	**	*	–	**	**
Levkovich and Omar (82)	Suicide Risk	2018–2024	7	29	–	**	–	**	–	–	*	*
Omar et al. (34)	Demographic Disparities	2018–2024	5	24	**	**	*	**	–	–	*	*
Schwabe et al. (35)	Data Quality	1993–2024	3	120	*	**	*	**	*	–	**	*
Das et al. (83)	Security & Privacy	–	–	–	**	–	*	**	–	–	*	**
Chang et al. (37)	LLM Evaluation	2020–2023	–	–	**	–	*	**	–	–	**	**
Ong et al. (84)	Ethical & Regulatory	2020–2023	2	58	*	*	–	*	*	–	*	**
Ullah et al. (41)	Diagnostic Challenges	2020–2023	5	7	*	–	**	**	*	–	**	**
Lyu et al. (85)	Model Interpretability	2015–2022	5	–	–	–	*	**	–	–	**	*
Pool et al. (86)	Telehealth Ethics	2023	6	20	*	*	*	**	**	–	**	**
Wang et al. (36)	Conversational LLMs	2022–2023	3	65	*	**	*	**	–	–	**	**
Qin and Tong (40)	Primary Care LLMs	–	–	–	**	–	–	–	**	–	**	**
Pressman et al. (39)	Surgical Ethics	2023	5	53	**	**	*	**	*	–	**	**
Haltaufderheide and Ranisch (38)	LLM Ethics	2023	6	53	*	**	*	**	*	–	**	**
Current Study	Healthcare & Medicine	**	**	27	**	**	**	**	**	**	**	**

SCALE: –, No; *, PARTIALLY; **, YES; Y, Year; D, Databases; NPR, number of papers reviewed; P/B, preliminary/background; SLR, systematic literature review; RSA, related survey analysis; SO, survey objectives; ER&F, ethical regulation & frameworks; EPRP, ethical policies & research protocols; SR, significant results; FD, future directions; LLM, large language models.

Shool et al. (33) reviewed 761 studies on LLM performance in clinical medicine, offering a quantitative analysis of evaluation parameters, model usage, and specialty coverage. It reveals inconsistencies in evaluation methods, biases, and underrepresentation of medical-domain LLMs, highlighting a lack of standardization and ethical analysis. Similarly, Levkovich and Omar (82) synthesized findings from 29 investigations (2018–2024) on LLM applications for suicide prevention, detection, and risk assessment, aggregating diverse data for robust insights. It notes heterogeneity in evaluation methods, limited ethical discussion, and potential biases requiring further inquiry. Extending this examination of bias (34) systematically detailed demographic bias types in medical LLMs, detailing bias types, measurement methods, and mitigation strategies. While it provides strong insights, it lacks sufficient consideration of non-binary identities and ethical regulations in LLM applications.

Schwabe et al. (35) proposed the METRIC framework—a multi-dimensional data-quality approach based on 120 studies—to enhance trustworthy AI through systematic dataset evaluation. Although it offers a comprehensive categorization of awareness dimensions, it lacks explicit measurement guidelines, deep engagement with ethical policy, and thorough examination of large language models. Likewise, Das et al. (83) surveyed LLM security and privacy challenges, detailing vulnerabilities, attacks, and defense strategies, but its literature selection methodology was not rigorously described, and healthcare-specific concerns were inadequately addressed. Chang et al. (37) provided an extensive taxonomy of LLM evaluation methods across tasks, benchmarks, and protocols— including ethical aspects—but its limited focus on healthcare applications and absence of a systematic literature methodology reduce its utility for healthcare-specific ethical analysis.

In another study, Ong et al. (84) discussed ethical and regulatory challenges of LLMs in medicine, advocating for robust frameworks for responsible integration and offering actionable insights; however, it provided minimal empirical analysis and only cursory attention to healthcare-specific issues. Similarly, Ullah et al. (41) conducted a scoping review of challenges in implementing LLMs in digital pathology, synthesizing prior reviews but remaining focused on diagnostic medicine rather than broader healthcare ethics. Lyu et al. (85) mapped literature on biomedical language-model interpretability, presenting a taxonomy of technical challenges and research gaps, yet it lacked discussion of broader ethical considerations and healthcare-specific contexts.

Pool et al. (86) examined the responsible use of LLMs in telehealth using a concept matrix based on EU and Australian ethics guidelines, offering insights into ethical challenges and future directions; however, its scoping nature limited empirical depth and overlooked broader healthcare ethics. Wang et al. (36) synthesized 65 studies on conversational LLM applications in healthcare, categorizing use cases and ethical concerns such as reliability, bias, privacy, and acceptability, and suggesting future research. Their analysis primarily focused on ChatGPT and noted variable study quality with limited exploration of complex ethical issues. Qin and Tong (40) reviewed LLM applications in primary health care, emphasizing opportunities and the need for robust ethical–legal frameworks, but lacked a systematic methodology and quantitative analysis.

Finally, Pressman et al. (39) reviewed 53 surgical studies to identify ethical concerns and core principles in LLM use, offering insights into accuracy, bias, and verifiability. However, its focus on 2023 publications and variable study quality limits broader policy implications. Haltaufderheide and Ranisch (38) synthesized 53 investigations on ChatGPT and LLMs in healthcare, mapping ethical challenges such as fairness, bias, non-maleficence, disclosure, and privacy; while it provides quantitative insights through comparative tables, its reliance on 2023 data and preprint sources limits geographic diversity and generalizability.

Our review focuses on the ethical implications of large language models (LLMs) in healthcare, specifically addressing patient privacy, bias mitigation, output explainability, accountability, and governance. By providing a targeted synthesis, it fills a crucial gap in guidance for responsible integration into clinical workflows and enables precise evaluation of risks and benefits in life-critical contexts. The insights generated are directly actionable for practitioners, developers, and policymakers committed to safeguarding patient safety, equity, and trust.

## 4 Methodology

**Figure 2:**
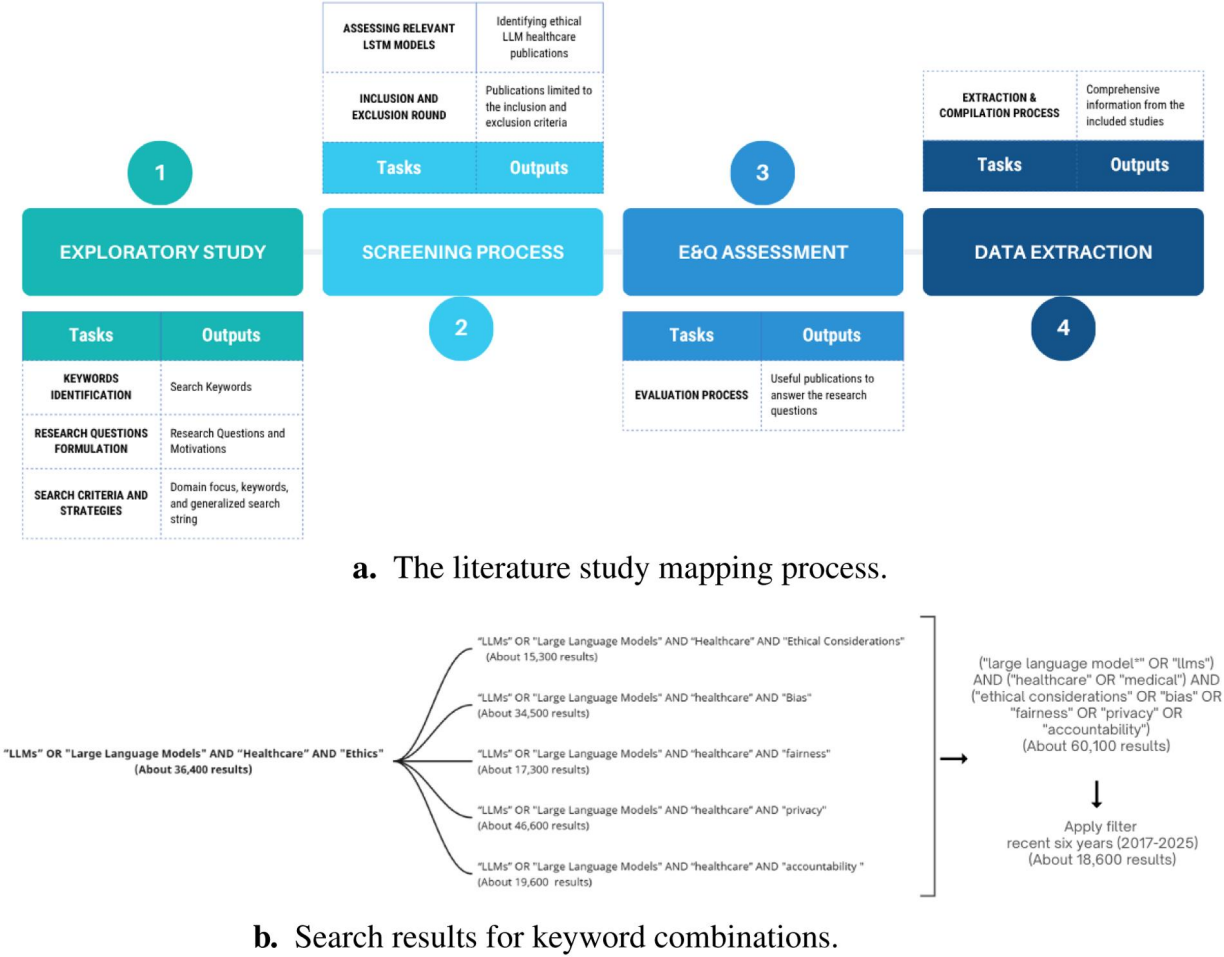
Overview of the study identification phase. **(a)** The literature study mapping process. **(b)** Search results for keyword combinations.

This review follows the Preferred Reporting Items for Systematic Reviews and Meta-Analyses (PRISMA) 2020 to ensure clarity and completeness in systematic reviews (42), and adheres to the guidelines by Kitchenham and Charters (2007) for systematic literature reviews (43). The study employs a four-phase process (illustrated in Figure 2a) to gather, analyze, and synthesize literature on the ethical considerations of large language models (LLMs) in healthcare. The phases include Phase 1: Exploratory study (Section 4.1), which covers keyword identification (Section 4.1.1), formulation of research questions (Section 4.1.2), and search criteria establishment (Section 4.1.3); Phase 2: Screening process (Section 4.2), involving literature inclusion and exclusion and the assessment of ethical dimensions in LLM deployment; Phase 3: Eligibility and quality assessment (Section 4.3), applying predefined criteria to full-text articles for rigorous evaluation; and Phase 4: Data extraction and compilation (Section 4.4), where bibliographic details and ethical variables are recorded for synthesis.

### 4.1 Phase 1: exploratory study

This is the initial stage of this systematic review, aiming to establish a foundation for analyzing the ethical considerations of LLMs in healthcare. This phase starts with an exploratory survey of literature to understand LLM applications in clinical settings and the discussed ethical dimensions (e.g., privacy, bias, transparency, accountability). Key activities include refining keywords related to LLMs, healthcare contexts, and ethical issues; iteratively improving the review framework; and defining precise search criteria and strategies (e.g., including terms like “large language model,” “healthcare,” “privacy,” “fairness,” “explainability”) across selected databases. This groundwork ensures that subsequent screening and analysis capture the most relevant literature on LLM ethics in healthcare.

#### 4.1.1 Keywords identification

In the initial phase of our review, we conducted an exploratory search using the string “large language models OR LLMs AND healthcare AND ethics” to gauge relevant literature. The preliminary results revealed various synonymous and related terms—such as GPT, BERT, Claude, medical, clinical, bias, fairness, privacy, and accountability—that frequently appear in pertinent study titles and abstracts. These keywords informed the refinement of our final search string, ensuring comprehensive coverage of large language model research in healthcare with a focus on ethical considerations. Example outcomes of selected keyword combinations are illustrated in Figure 2b.

#### 4.1.2 Research questions formulation

In this section, we refined the research questions (RQs) to focus on critical aspects of ethical considerations surrounding the use of large language models (LLMs) in healthcare, addressing key knowledge gaps identified during our initial literature survey. The research questions are as follows:

1. **RQ1: What are the main ethical issues of Large Language Models in healthcare?** (see Section 5.1)
  Motivation: to document key ethical challenges arising from LLM deployment in clinical settings and assess their implications for patient safety and equity
2. **RQ2: Which LLM architectures are most frequently employed in healthcare ethics studies?** (see Section 5.2)
  Motivation: to survey prevalent model families and frameworks used in ethical analyses, identifying trends that shape methodological rigor and reproducibility
3. **RQ3: Which healthcare application domains have been most frequently examined in ethical analyses of LLMs?** (see Section 5.3)
  Motivation: to map the focus areas of ethical inquiry across medical contexts, highlighting domains with concentrated scrutiny and those needing further attention
4. **RQ4: What publication and bibliographic patterns characterize the literature on ethical considerations of LLMs in healthcare?** (see Section 5.4)
  Motivation: to examine dissemination venues, temporal trends, and bibliometric features that inform where and how ethical research on LLMs is shared

#### 4.1.3 Search criteria and strategies

**Figure 3:**
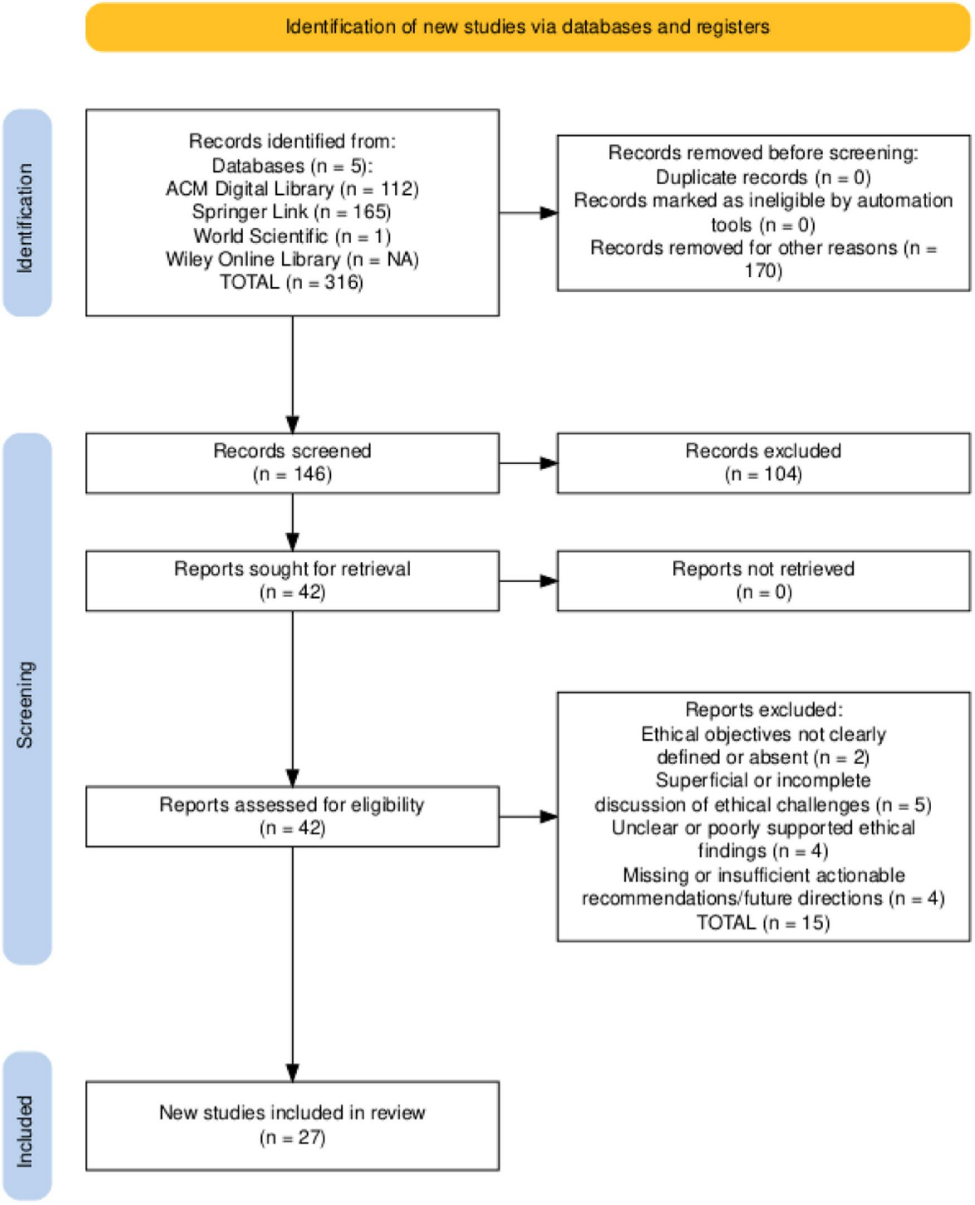
PRISMA flowchart of the systematic literature process.

To ensure comprehensive coverage of ethical discussions on large language models (LLMs) in healthcare, we searched five scientific databases, ACM Digital Library, Springer Link, World Scientific, Wiley Online Library, and PubMed, for publications from January 2017 to June 2025. The final search on June 10, 2025, used advanced queries combining keywords (e.g., “large language models OR LLMs” AND “healthcare” AND “ethics”) with logical operators AND/OR and relevant wildcards. The initial search yielded 316 records, which we imported into a reference manager, removed duplicates from, and screened titles and abstracts following PRISMA guidelines (42). We predefined inclusion and exclusion criteria to focus on peer-reviewed studies that address the ethical dimensions of LLM deployment in clinical contexts. The complete search syntax, date range, and inclusion/exclusion metrics are provided in the supplementary Excel file “*Table 1 – Search String*”, enabling cross-verification of each URL, query, and retrieval count. A PRISMA flowchart documents the selection process (see Figure 3), ensuring traceability and reproducibility.

### 4.2 Phase 2: screening process

We retrieved 316 records from five databases and applied an initial filter to exclude paid-access articles, reviews, surveys, commentaries, and other non-relevant formats. This resulted in 146 research papers for title and abstract screening. We then rigorously evaluated these papers against predefined inclusion and exclusion criteria (outlined in Table 4), focusing on the ethical considerations of LLMs in healthcare. We excluded literature that did not meet these criteria or lacked a clear emphasis on LLM ethics in clinical contexts. Consequently, 146 research papers remained for full-text assessment. Detailed stage-wise metrics for all databases appear in the “*Search String*” sheet of the supplementary file “*Table 1*”, ensuring reproducibility and allowing assessment of potential database- and language-based biases.

**Table 4:** Criteria for selecting studies for systematic review.

Criteria	Inclusion criteria	Exclusion criteria
Year of Publication	2017 to 2025	Outside 2017–2025
Type of Article	Journal papers, conference proceedings, book chapters	Review papers, tutorials, seminars, interviews, letters, blogs
Language	English	Non-English
Text Availability	Full-text available	Abstracts only or incomplete texts (<5 pages)
Relevance to RQs	Directly relates to the RQs	Irrelevant to the RQs
Publication Status	Published	Unpublished or in press
Access	Open access	Restricted access or subscription required
Study Design	Empirical evidence, theoretical frameworks, or case studies on LLMs in healthcare	Non-peer-reviewed articles, opinion pieces, editorials, or speculation
Focus Area	Large language models in healthcare	Studies not related to healthcare or LLMs
Ethical Focus	Addresses safety, trust, security, privacy, bias, transparency, accountability, or responsible LLM use	Articles not addressing AI ethics

### 4.3 Phase 3: eligibility and quality assessment (EQA)

**Figure 4:**
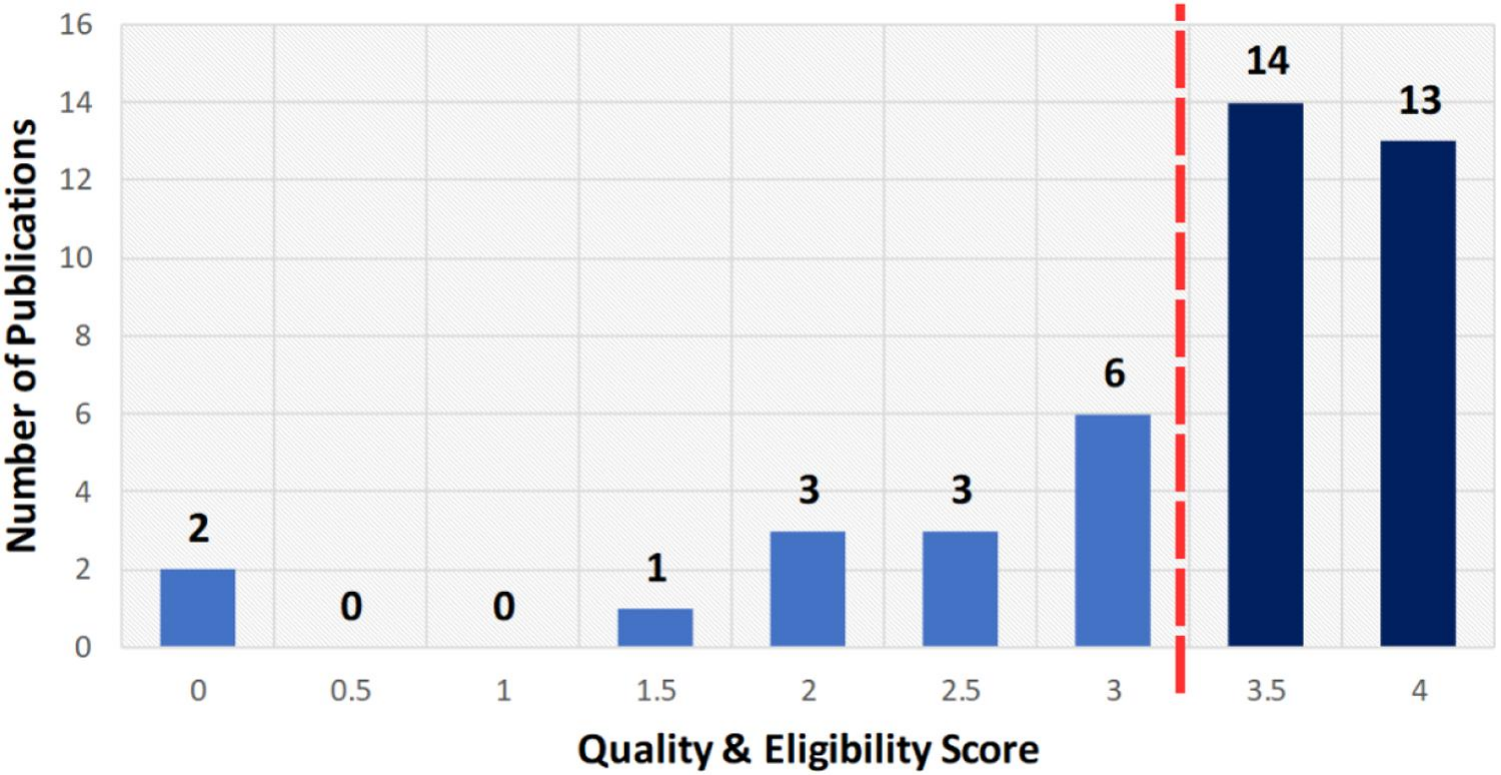
Quality score distribution of studies.

After screening the titles and abstracts, 42 papers underwent a detailed eligibility and quality assessment to ensure the inclusion of only robust and relevant papers. Each research paper was evaluated using the scoring system in Table 5 on a scale from 0 to 4, where 0 indicates non-compliance, 0.5 indicates partial compliance, and 1 indicates full compliance. Criteria included clearly defined ethical objectives, methodological rigor in LLM ethics, acknowledgement of limitations, and clarity of ethical findings. Studies scoring at least 3.5 (87.5%) (see Figure 4) were retained, resulting in 27 high-quality papers. This process confirmed that the included literature meets rigorous standards and directly addresses ethical considerations of LLM deployment in healthcare. Each full text was scored independently by three reviewers using the criteria in Table 5; disagreements were resolved by discussion. Our initial independent scores agreed on over 90% of the criteria before reconciliation. We did not calculate Cohen’s kappa, but all discrepancies were reconciled through consensus. This procedure follows PRISMA 2020 and Kitchenham & Charters 2007 (42, 43).

**Table 5:** Criteria for scoring the eligibility and quality assessment.

Criteria	Score	Description
Objectives Clearly Stated?	1	Ethical objectives and goals are clearly defined and focused on LLM deployment in healthcare.
	0.5	Ethical objectives are mentioned but lack precise definitions or clear linkage to LLM use.
	0	Ethical objectives are unclear or absent.
Ethical Challenges Discussed?	1	Ethical challenges are thoroughly identified and contextualized for LLM applications in healthcare.
	0.5	Some challenges are mentioned, but the analysis is superficial.
	0	No meaningful discussion of ethical challenges.
Ethical Recommendations or Future Directions?	1	Provides actionable recommendations and future research directions for ethical LLM integration in healthcare.
	0.5	Offers some recommendations, but lacks depth or relevance.
	0	No recommendations or future directions are included.
Clarity of Ethical Findings?	1	Ethical findings are clear, well-supported, and effectively communicated.
	0.5	Findings are presented but lack clarity and sufficient evidence.
	0	Findings are unclear or absent.

### 4.4 Phase 4: data extraction and compilation

Data from the 27 selected papers were extracted into a structured spreadsheet. For each paper, we recorded core metadata, including Name of Database, Publisher, Journal or Conference, Publication Type, Author, Title, Abstract, Keywords, Year, DOI, URL, and, when applicable, Volume, Issue, Pages, and Month. We also extracted ethics-specific variables such as Ethical Contributions, Application Area, Ethical Policies, LLM Model Discussed, Ethical Objectives, Ethical Challenges, Sub-Ethical Challenges, Law/Regulation/Frameworks, Key Ethical Findings, and Future Directions. This process ensured a comprehensive capture of bibliographic information and relevant details. A cross-verification step validated the consistency and accuracy of all extracted fields. Table 6 summarizes the number of papers retrieved and included at each stage of the review process.

**Table 6:** Studies retrieved in each step of the systematic literature process.

Process	Retrieved	Identified	Screened	Pass E&QA	Included
ACM	112	61	22	16	16
Springer Link	165	54	7	2	2
World Scientific	1	1	0	0	0
Wiley Online Library	17	9	2	1	1
PubMed	21	21	11	8	8
TOTAL	316	146	42	27	27

## 5 Results and discussion

This section synthesizes findings from the 27 included papers to address the research questions. We provide a concise overview of the primary ethical issues, prevalent model families, application domains, and publication patterns in LLM research for healthcare through visual representations and data summaries.

### 5.1 RQ1: what are the main ethical issues of large language models in healthcare?

**Figure 5:**
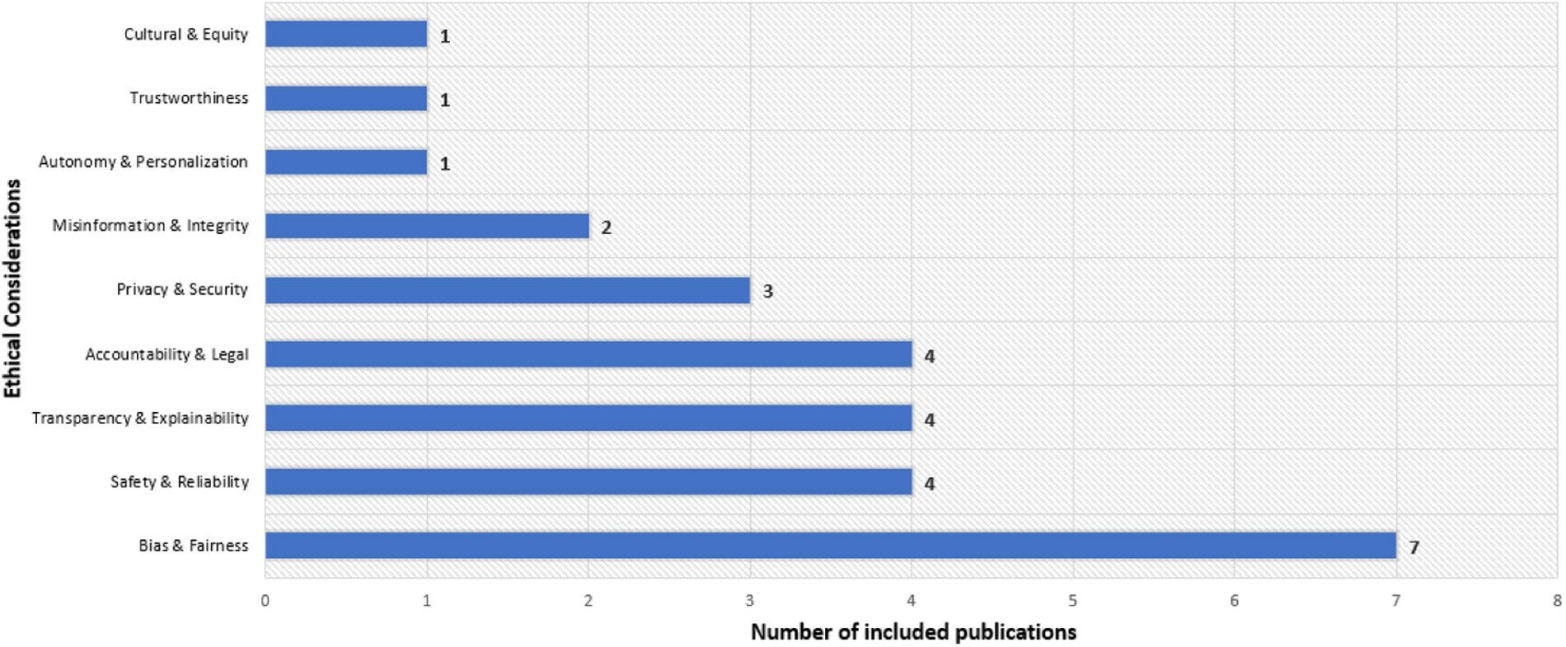
Distribution of included studies by ethical issues.

Recent research studies identify various ethical concerns related to large language models in healthcare. The most frequent issue is bias and fairness (n=7) (7, 8, 10, 13, 15, 17, 27), followed by safety and reliability (n=4) (20, 22, 24, 70), transparency and explainability (n=4) (9, 14, 21, 32), and accountability and legal issues (n=4) (25, 26, 31, 71) (see Figure 5). Privacy and security (n=3) (12, 23, 67) and misinformation and integrity (n=2) (16, 72) also receive attention, while autonomy and personalization (19) and cultural and equity issues (18) appear less frequently (n=1 each). This highlights a focus on bias mitigation and system reliability while noting gaps in model governance and inclusivity. The following sections provide summaries and detailed tables for each ethical category.

#### 5.1.1 Bias and fairness

Several research papers have evaluated methods to uncover and reduce bias in healthcare language models. One study presents a model-agnostic approach to detect and mitigate gendered language in clinical notes, showing that targeted data augmentation can reduce gender bias without degrading classification performance and provides interpretable insights for healthcare text analytics (15). Another investigation quantifies disparities in Clinical BERT embeddings across gender, language, ethnicity, and insurance status, revealing persistent performance gaps in predictive tasks. Attempts at adversarial debiasing only partially alleviate these issues, highlighting the need for more effective mitigation strategies (17). In the mental health domain, MentaLLaMA is introduced as an open-source, instruction-tuned model fine-tuned on a specialized dataset to generate human-level explanations with predictions, enhancing both accuracy and interpretability for sensitive applications (13). Additionally, an empirical research with autistic individuals shows how LLMs can assist in reframing negative self-talk while risking the reinforcement of neurotypical biases, offering design insights for neuro-affirming, personalized AI tools that complement traditional therapy (7).

Other work examines biases in widely used commercial models and their broader implications. Analysis of GPT-4 outputs for diagnostic and treatment planning reveals significant demographic biases, such as skewed prevalence estimates and differential recommendations, which may exacerbate health disparities. This situation calls for rigorous bias assessments and tailored mitigation before clinical deployment (27). Evaluations of several commercial LLMs in medical education show inconsistent and sometimes racist content when querying debunked race-based medicine scenarios, posing risks for clinical decision-making. Authors advocate for extensive red teaming, data provenance documentation about training data, and larger quantitative research papers to address these biases (10). Finally, applying Schwartz’s value theory to compare Bard, Claude.AI, GPT-3.5, and GPT-4 reveals Western-centric value orientations in model outputs, influencing clinical recommendations and highlighting the need for cultural calibration and thorough vetting to ensure equitable AI-assisted decision-making (8). Table 7 summarizes the applications, LLMs, ethical issues, and key findings related to bias and fairness.

**Table 7:** Summary of key ethical concerns in LLM healthcare studies.

Study	Application area	LLM model discussed	Ethical issues	Key ethical findings
Minot et al. (15)	Clinical text debiasing	BERT, Clinical BERT	Bias & fairness	Data augmentation reduces gender bias in clinical notes while preserving classification accuracy.
Zhang et al. (17)	Condition prediction modeling	BERT, SciBERT, Clinical BERT	Bias & fairness	Clinical BERT embeddings reveal demographic performance gaps; adversarial debiasing only partially mitigates disparities.
Yang et al. (13)	Mental health explanation	MentaLLaMA, LLaMA2	Bias & fairness	Domain-specific fine-tuning achieves accurate and interpretable mental health predictions.
Carik et al. (7)	Autism mental health support	ChatGPT, Claude	Bias & fairness	LLMs can support autistic self-talk reframing but risk neurotypical bias, highlighting need for personalization.
Zack et al. (27)	Diagnostic and treatment planning	GPT-4	Bias & fairness	GPT-4 outputs exhibit demographic bias in prevalence estimates and recommendations, risking exacerbation of health disparities.
Omiye et al. (10)	Medical education enhancement	ChatGPT (GPT-4), Bard, Claude	Bias & fairness	Commercial LLMs generate inconsistent, race-biased medical responses, posing risks for clinical decision-making.
Hadar-Shoval et al. (8)	Clinical ethics assessment	Bard, Claude, GPT-3.5, GPT-4	Bias & fariness	LLMs encode Western-centric value orientations that influence clinical recommendations, indicating cultural bias.
Choi et al. (20)	Eating disorder support	GPT-4	Safety & reliability	Unquestioning user trust risks unsafe recommendations.
Pope and Patooghy (24)	FHIR standard comprehension	GPT-3.5, GPT-4	Safety & reliability	Insufficient accuracy threatens data integrity and patient safety.
Lee et al. (22)	Patient navigation planning	GPT-4	Safety & reliability	Formal verification mitigates hallucinations and improves reliability.
Cheong et al. (70)	Generative AI governance	General LLMs	Safety & reliability	Existing legal frameworks fail to mitigate opaque AI harms.
Marvin et al. (14)	Symptom-based diagnostics	BERT	Transparency & explainability	Model-agnostic XAI methods enable interpretable diagnostics and support regulatory transparency.
Kitamura et al. (32)	Explainable AI in medicine	ChatGPT	Transparency & explainability	Code-based prompts enable verifiable, transparent decision-makingvs. opaque standard prompts.
Fiori et al. (21)	Smart home health monitoring	GPT-3.5, GPT-4	Transparency & explainability	Automated evaluation of explanations aligns with user judgments, supporting reliable and fair model assessment.
McBain et al. (9)	Suicide prevention evaluation	ChatGPT-4o, Claude, Gemini	Transparency & explainability & Explainability	LLMs systematically overestimate appropriateness of suicide intervention responses, posing ethical risks.
Yigit et al. (26)	Emergency medicine triage	GPT-4	Accountability & legal & Privacy	Critical omissions risk patient safety and accountability.
Ye et al. (31)	Clinical documentation support	ChatGPT	Accountability & legal	Omissions in AI-generated reports risk misinterpretation without clinician verification.
Wang et al. (25)	Radiology and discharge summaries	ChatGPT (GPT-4)	Accountability & legal	Liability ambiguity and biased, non-transparent outputs threaten care integrity.
Kavian et al. (71)	Surgical decision support	ChatGPT	Accountability & legal	Safety lapses and unclear accountability require clinician oversight and safeguards.
Liu et al. (12)	Federated LLM fine-tuning	GPT-4, LLaMA-7B	Privacy & security	DP-LoRA ensures strict differential privacy with reduced communication overhead.
Montagna et al. (23)	Chronic disease management	GPT-4	Privacy & security & Access Control	Decentralized architecture grants patient control over data, mitigating privacy risks.
Jo et al. (67)	Voice-based health chatbots	HyperCLOVA	Privacy & security	Long-term memory enhances engagement but raises privacy concerns requiring careful management.
Upadhyay et al. (16)	Fake health news detection	BERT, BioBERT, BERTweet-Covid	Misinformation	Socio-contextual features improve fake health news detection accuracy and transparency.
Angelis et al. (72)	Scientific writing assistance	ChatGPT	Research Integrity	Rapid text generation risks fueling an infodemic and undermining research integrity.
Arum et al. (19)	Physical activity coaching	GPT-4.0	Autonomy, Transparency & Personalization	Alignment with user decision styles is essential for trust and effective health recommendations.
Antoniak et al. (18)	Maternal healthcare chatbots	GPT-3.5	Power Imbalance, Equity	Inclusive design must address safety, privacy, and historical inequities for equitable maternal healthcare.
Chen et al. (11)	Ophthalmology patient interaction	EyeGPT (LLaMA2-7b-chat)	Hallucinations, Trustworthiness & Empathy	Domain-specific fine-tuning and retrieval augmentation reduce hallucinations and enhance trustworthiness and empathy.

#### 5.1.2 Safety and reliability

Recent analyses show both benefits and risks when deploying LLM tools in healthcare. An investigation of an eating disorder recovery chatbot finds that WellnessBot can empower users but may lead them to trust suggestions without critique, risking unsafe advice, so designs must encourage critical engagement and oversight (20). In interoperability settings, evaluations of LLMs with FHIR standards show they do not meet accuracy thresholds for clinical tasks, raising data integrity and patient safety concerns, and recommend domain-specific evaluation protocols, calibration strategies, and reliability checks before integration (24). In planning scenarios, combining formal verification with LLM outputs reduces hallucinations and constraint violations, indicating that user-driven rule checks can boost trust and output quality in safety-critical tasks such as patient navigation (22). A governance analysis finds current legal frameworks insufficient to prevent harms such as biased outputs and privacy breaches because of model opacity, and proposes a Responsible AI Legal Framework that embeds human values into enforceable guidelines for healthcare (70). These findings suggest that safe and effective LLM use in healthcare requires user-centered design, rigorous evaluation, formal verification, and legal reform. Table 7 summarizes the applications, LLMs, ethical issues, and key findings related to safety and reliability.

#### 5.1.3 Transparency and explainability

Marvin et al. (14) integrates model-agnostic explanation techniques to interpret complex symptom-based diagnostic models, supporting accountability and regulatory compliance in digital health systems. Another study introduces a code-based prompting approach to reveal the logic behind medical decision outputs, showing that standard prompting remains opaque and emphasizing the need for verifiable workflows to build user confidence (32). In smart home health monitoring, automated evaluation of explainable AI methods aligns with user judgments, offering a scalable alternative to manual surveys for assessing explanation quality and promoting transparency (21). In mental health applications, comparing LLM-generated ratings to expert suicidologists reveals a systematic upward bias, indicating ethical risks if deployed without expert benchmarking and highlighting the need for human-in-the-loop alignment, rigorous benchmarking, and reinforcement learning from expert feedback to ensure safe support for individuals with suicidal ideation (9). These insights underscore the role of transparent evaluation, explanation mechanisms, and expert oversight in responsible LLM deployment in healthcare. Table 7 summarizes the applications, LLMs, ethical issues, and key findings related to transparency and explainability.

#### 5.1.4 Accountability and legal

Yigit et al. (26) highlights the promise of LLM tools alongside necessary safeguards in clinical settings. In emergency triage for mild traumatic brain injury, GPT-4 can offer clear guidance but may omit critical details and pose readability challenges that threaten patient safety and accountability, underscoring the need for clinician oversight in acute care. Using ChatGPT to draft clinical case reports can streamline workflows, but omissions of key patient history mean clinicians must review AI outputs carefully to prevent misinterpretation and maintain data integrity (31). Analyses in radiology and surgery further emphasize legal considerations. In radiology and discharge summary generation, ChatGPT raises concerns about liability ambiguity, reduced compassionate care, algorithmic bias, and content validity, indicating an urgent need for clear regulations, robust validation, and measures to protect patient trust (25). In surgical decision support, AI may aid diagnostics and postoperative planning, but safety lapses, privacy risks, and unclear responsibility require secondary clinician review, explicit disclaimers distinguishing AI suggestions from clinician judgments, strong data de-identification protocols, and informed consent processes for responsible integration (71). Table 7 summarizes the applications, LLMs, ethical issues, and key findings related to accountability and legal.

#### 5.1.5 Privacy and security

Approaches show how privacy-preserving architectures enable collaborative or personalized LLM healthcare services without exposing sensitive data. A federated fine-tuning method introduced by Liu et al. (12) integrates differential privacy with low-rank adaptation, allowing institutions to improve domain-specific models while preventing data leakage and reducing communication overhead in distributed settings. A decentralized chatbot design uses personal data stores and smart contracts to give patients control over their health records, aligning technical choices with ethical imperatives and regulatory requirements for chronic disease management (23). In voice-based health chatbots, incorporating long-term memory enhances engagement and self-disclosure but raises privacy concerns when recalling sensitive details, highlighting the need for careful memory curation and robust management strategies (67). These studies highlight the tension between utility and confidentiality and call for refining privacy mechanisms and governance measures to support trust in LLM-driven healthcare applications. Table 7 summarizes the applications, LLMs, ethical issues, and key findings related to privacy and security.

#### 5.1.6 Other ethical issues

Upadhyay et al. (16) integrates socio-contextual signals into a BERT-based detector for fake health news, showing that context features improve explainability via SHAP and strengthen trust in filtering systems. Another warns of an “AI-driven infodemic,” noting that unregulated model outputs may flood scientific literature with misleading content, and calls for detectable-by-design text and expert oversight to protect research integrity (72). Research on personalization and equity highlights ethical tensions in tailored health support. An AI-assisted activity coaching study finds that aligning recommendations with individual decision styles is vital for trust and autonomy, suggesting transparent personalization mechanisms to avoid undermining user confidence (19).

In maternal healthcare, participatory design workshops produce guiding principles that emphasize inclusiveness and historical context to mitigate power imbalances and ensure equitable NLP tool development (18). Work on clinical LLM assistants shows that domain-specific fine-tuning and retrieval augmentation can reduce hallucinations and enhance empathy in patient interactions, but requires rigorous validation and ongoing refinement to maintain safety and trustworthiness in diverse real-world settings (11). Table 7 summarizes the applications, LLMs, ethical issues, and key findings related to other ethical issues.

### 5.2 RQ2: which LLM architectures are most frequently employed in healthcare ethics studies?

**Figure 6:**
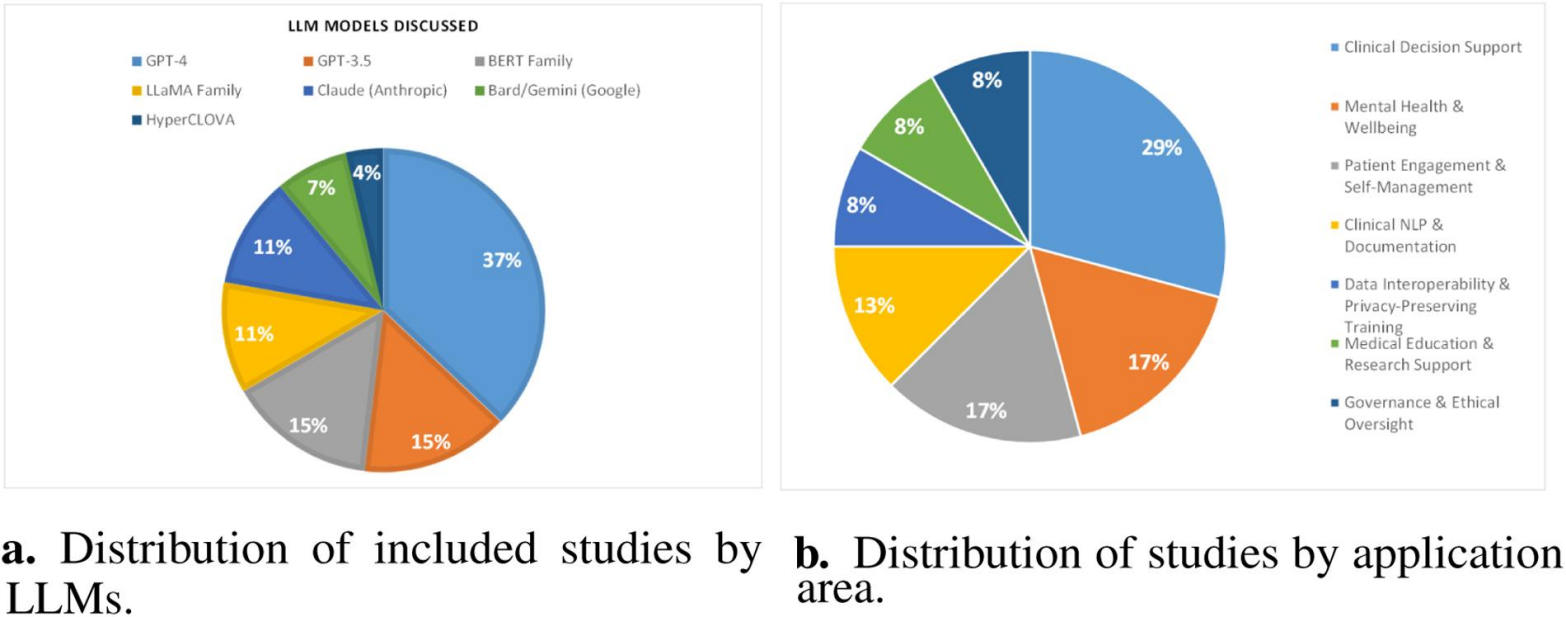
Overview of model usage and application domains. (**a**) Distribution of included studies by LLMs. (**b**) Distribution of studies by application area.

Among 27 studies, GPT-4 variants appear in ten papers (8, 10, 12, 20, 22–27), highlighting their prominence in ethical analyses. Unspecified ChatGPT appears in five (7, 31, 32, 71, 72), reflecting interest in conversational interfaces without precise versions. GPT-3.5 models occur in three studies (8, 18, 24). BERT-family architectures feature in four (14–17), showing continued attention to encoder-based approaches. LLaMA variants appear in three papers (11–13). Claude is examined in four (7–10), indicating interest in alternative offerings. Bard and Gemini appear in studies (8–10), while HyperCLOVA is addressed in one (67). General references to LLMs occur in a single paper (70). This distribution shows a clear focus on widely adopted generative models alongside diverse architectures, suggesting that ethical debates often center on popular platforms but also consider a broad range of systems. Figure 6a shows the distribution of studies by LLMs.

### 5.3 RQ3: which healthcare application domains have been most frequently examined in ethical analyses of LLMs?

An analysis of the selected corpus shows that clinical decision support is the most examined domain, appearing in seven papers (14, 17, 22, 26, 27, 32, 71), indicating a strong focus on integrating LLMs into diagnostic and treatment workflows. This scoping review (87) examines ethical and legal challenges of deploying LLMs in emergency medicine, emphasizing the critical need for explainable AI to ensure patient safety and compliance with global data protection standards amidst heterogeneous adoption patterns. Mental health and wellbeing contexts follow with four papers (7, 9, 13, 20), reflecting attention to sensitive psychological applications. Patient engagement and self-management appear in four papers (18, 19, 23, 67), underscoring efforts to empower individuals via chatbots and coaching tools. Data interoperability and privacy-preserving fine-tuning emerge in two studies (12, 24), highlighting technical and policy challenges around data sharing. Clinical NLP and documentation ethics are addressed in three studies (15, 25, 31), pointing to concerns in text processing and record generation. Medical education and research support appear in two papers (10, 72), while governance and ethical oversight feature in two (8, 70). Public health informatics and misinformation are represented by this study (16), and specialty-specific patient interaction by this one (11). Figure 6b illustrates the application area-wise distribution of included papers.

### 5.4 RQ4: what publication and bibliographic patterns characterize the literature on ethical considerations of LLMs in healthcare?

Analysis of reviewed papers reveals clear bibliographic trends: publications increase from 2020 to 2025, and sources vary across major databases and publishers. This overview identifies which venues address LLM ethics most often, the balance between journal articles and conference papers, and concentrations in specific journals or publishers. Examining these patterns pinpoints active outlets for ethical discussions and uncovers gaps in dissemination. Overall, this synthesis offers insight into the field’s maturity and focus areas, guiding authors toward relevant outlets and informing future research strategies.

#### 5.4.1 Distribution of studies by databases

**Figure 7:**
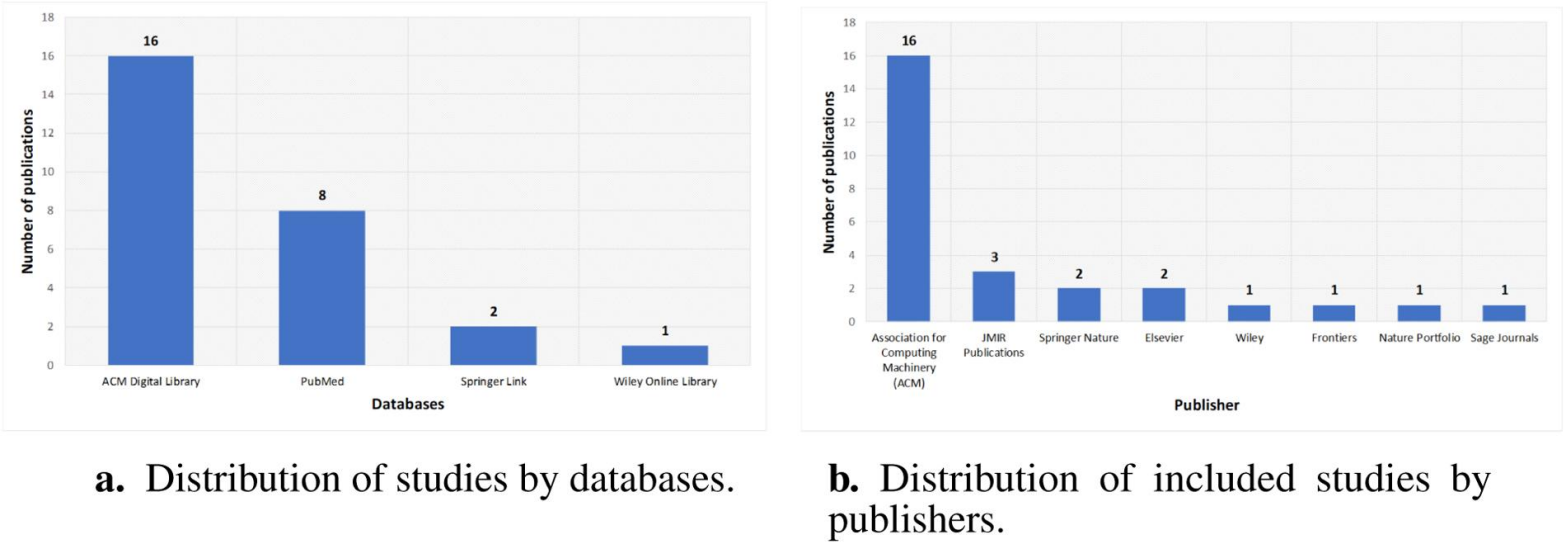
Bibliographic characteristics of included studies. (**a**) Distribution of studies by databases. (**b**) Distribution of included studies by publishers.

The distribution by database highlights primary sources for research on ethical considerations of LLMs in healthcare. ACM Digital Library contributes 16 publications, reflecting its prominence in technical and interdisciplinary AI ethics work. PubMed follows with 8 studies, indicating strong clinical and biomedical interest in LLM ethics. Springer Link and Wiley Online Library account for 2 and 1 publications, respectively, suggesting fewer but noteworthy contributions. As shown in Figure 7a, this spread indicates where ethical discussions are most frequently indexed and suggests potential gaps in other repositories.

#### 5.4.2 Distribution of studies by publisher

Publisher-wise investigation shows concentration in a few outlets for ethical research on LLMs in healthcare. The Association for Computing Machinery has 16 publications, reflecting its central role in AI ethics. JMIR Publications has 3 papers, showing clinical informatics interest, while Springer Nature and Elsevier each have 2 publications. Wiley, Frontiers, Nature Portfolio, and Sage Journals each contribute one publication, indicating diverse but limited representation. Figure 7b shows that ethical discussions on LLM deployment in healthcare appear mostly in these outlets and suggests opportunities to expand engagement elsewhere.

#### 5.4.3 Annual trends in research paper identification and inclusion

**Figure 8:**
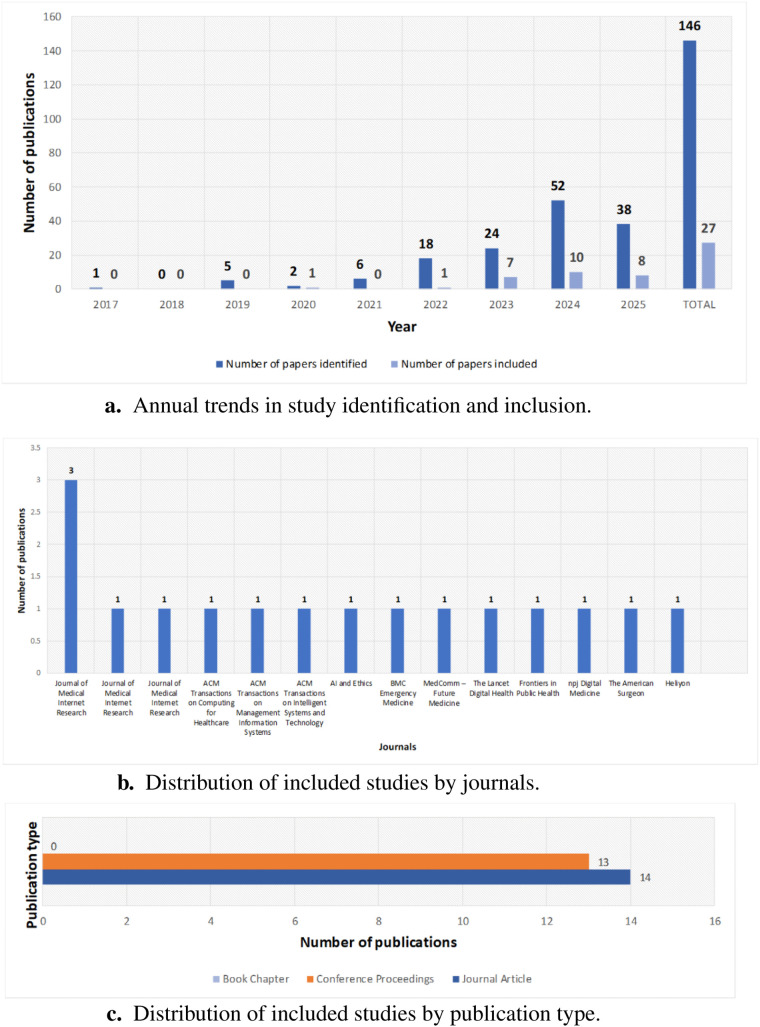
Publication and bibliographic patterns. (**a**) Annual trends in study identification and inclusion. (**b**) Distribution by journals. (**c**) Distribution by publication type.

A year-by-year breakdown shows a rise in identified papers on LLM ethics in healthcare, from a few records before 2020 to peaks of 52 in 2024 and 38 in 2025. Despite this increase, the share of meetings’ inclusion criteria remains modest: 1 of 2 in 2020, 1 of 18 in 2022, 7 of 24 in 2023, 10 of 52 in 2024, and 8 of 38 in 2025. Figure 8a illustrates annual trends in study identification and inclusion.

#### 5.4.4 Journal-wise distribution of publications

The distribution of papers across journals highlights key venues for research on LLM ethics in healthcare. Journal of Medical Internet Research leads with five publications. Other journals each contribute one paper, including ACM Transactions on Computing for Healthcare, ACM Transactions on Management Information Systems, ACM Transactions on Intelligent Systems and Technology, AI and Ethics, BMC Emergency Medicine, MedComm – Future Medicine, The Lancet Digital Health, Frontiers in Public Health, npj Digital Medicine, The American Surgeon, and Heliyon. Figure 8b shows this distribution, reflecting interdisciplinary engagement and growing interest across medical and computing journals.

#### 5.4.5 Publication type distribution

The breakdown of publication types, depicted in Figure 8c, shows a near-even split between journal articles (14) and conference proceedings (13), reflecting rigorous peer review and timely presentations on LLM ethics in healthcare. The absence of book chapters indicates emphasis on current research in rapidly evolving venues. This balance shows that the field values both journal depth and conference agility for emerging ethical insights.

## 6 Challenges, ethical framework and future prospects

### 6.1 Challenges

Although the reviewed papers yield valuable insights into ethical aspects of LLMs in healthcare, only 9 explicitly reference any legal or ethical frameworks [e.g., IRB protocols (11, 20), GDPR/HIPAA compliance designs (23), calls for institutional guidelines (25), or proposals for new governance models (8, 9, 14, 70, 72)]. This gap suggests limited grounding in regulatory structures. Empirical evaluations of explainability tools and accountability mechanisms remain scarce, and few assess long-term impacts on patient safety or equity. Methodological heterogeneity and lack of standardized ethical appraisal criteria hinder comparison across studies. These limitations highlight the need for stronger integration of formal governance considerations and standardized evaluation approaches when investigating LLMs in clinical contexts.

While algorithmic bias mitigation shows conceptual promise (35), most approaches lack real-world clinical validation. Similarly, transparency techniques like explainable AI (XAI) remain predominantly theoretical, with few large-scale evaluations in healthcare settings (32). These gaps contrast with significant advances in privacy-preserving architectures (83), revealing an imbalanced maturation of ethical safeguards across domains.

### 6.2 A provisional ethical integration framework for LLMs in healthcare

Building on our thematic synthesis of 27 studies, we propose a four-layer framework for ethically integrating LLMs into clinical contexts:

• **Regulatory Layer:** Legal and policy safeguards, including HIPAA/GDPR compliance, WHO and OECD AI ethics guidelines, and biomedical principlism, to ensure data-subject rights and enforceable governance (see Sections 2.4.1, 2.4.2, 2.4.3).
• **Technical Safeguards:** Algorithmic fairness and privacy-by-design measures such as bias audits, differential privacy, federated learning, and adversarial debiasing to mitigate harms at the system level (see Sections 5.1.1, 5.1.5).
• **Human Oversight:** Clinician-in-the-loop review, informed-consent procedures, and participatory design workshops that embed end-user expertise and agency in model development and deployment (see Sections 5.1.4, 5.1.6).
• **Transparency & Accountability:** Explainability techniques (e.g., model-agnostic methods, code-based prompting), audit trails, and red-teaming to promote trust, facilitate external review, and assign responsibility (see Section 5.1.3).

This framework synthesizes the ethical requirements identified across our reviewed studies into four interdependent domains, providing a clear roadmap for future theoretical development and empirical validation.

### 6.3 Future prospects

Drawing on our synthesis and proposed framework, we outline the following key thematic priorities for future research:

1. There is a clear call for systematic governance models and regulatory frameworks tailored to LLMs in healthcare, including Responsible AI Legal Frameworks that enshrine patient safety, privacy, and fairness as enforceable standards (25, 70, 71).
2. Methodological enhancements should include rigorous bias assessments and debiasing strategies across diverse demographic groups (10, 13, 15, 17, 27), stronger differential privacy mechanisms in federated training (12), and improved prompt engineering and calibration to boost reliability and reduce hallucinations (22, 24, 26).
3. Human-centered safeguards such as continuous monitoring, clinician oversight in high-stakes decisions, and participatory design processes will help align LLM outputs with clinical realities and user needs (7, 18, 20, 31).
4. Transparency efforts should go beyond local explainability techniques to include global interpretability and automated evaluation methods tailored to stakeholders (14, 21, 32).
5. While this review cites LLM-specific decision-support studies (19, 32, 71) and outlines broader ethical frameworks in Section 2.4.3, including (79)’s biomedical principles and Floridi’s information ethics (78), a systematic examination of the extensive general literature on human and assisted decision-making ethics remains to be undertaken. We recommend that future work conduct a dedicated, interdisciplinary synthesis, bridging LLM-centric research with foundational clinical-ethics scholarship, to develop more comprehensive decision-support guidelines and to validate them through empirical clinical studies.
6. Finally, context-specific adaptations—such as culturally sensitive calibrations (8), personalized recommendation alignment (19), and domain-focused fine-tuning with ethical guardrails—will be essential for responsible deployment.

These directions suggest a multi-pronged agenda combining policy development, technical rigor, and stakeholder engagement to strengthen the ethical integration of LLMs in healthcare.

## 7 Conclusion

While our thematic synthesis of 27 peer-reviewed papers identifies bias, safety, transparency, accountability, and privacy as dominant ethical considerations, these findings reflect only the selected corpus and should not be interpreted as a comprehensive representation of all ethical issues in LLM-deployed healthcare research. GPT-family models dominate current ethical analyses, particularly in clinical decision support, mental health, and patient engagement domains. The review underscores the growing need for regulatory frameworks, bias mitigation strategies, transparent model evaluation, and stakeholder-driven safeguards and introduces a provisional ethical integration framework to guide holistic LLM deployment.

### 7.1 Limitations

This analysis is constrained by its English-language, open-access corpus (2017–mid 2025), which may exclude significant non-English or subscription-based research. Our thematic approach, while systematic, relies exclusively on published literature and may overlook practical clinical insights. Additionally, the authors are not professionally trained as clinicians or ethicists; although two co-authors have formal coursework in professional ethics, and we sought advisory input from practicing clinicians, this does not replace dedicated clinical-ethics expertise. We anchored our analysis in established frameworks [e.g., World Health Organization (76), Beauchamp & Childress (79)], yet we did not exhaustively review the broader literature on human and assisted decision-making ethics. Future work should involve direct collaboration with professional clinicians and ethics specialists to ensure comprehensive ethical grounding.

## Data Availability

The raw data supporting the conclusions of this article will be made available by the authors, without undue reservation.
